# Mesoporous Zn/MgO Hexagonal Nano-Plates as a Catalyst for *Camelina* Oil Biodiesel Synthesis

**DOI:** 10.3390/nano11102690

**Published:** 2021-10-13

**Authors:** Lai-Fan Man, Tsz-Lung Kwong, Wing-Tak Wong, Ka-Fu Yung

**Affiliations:** 1 Department of Chemistry, The University of Hong Kong, Pokfulam Road, Hong Kong, China; h0992119@hku.hk; 2 Institute of Textiles and Clothing, The Hong Kong Polytechnic University, Hung Hom, Kowloon, Hong Kong, China; tlsamuel.kwong@polyu.edu.hk; 3 Department of Applied Biology and Chemical Technology, The Hong Kong Polytechnic University, Hung Hom, Kowloon, Hong Kong, China

**Keywords:** Zn/MgO, nano-plates, mesoporous, heterogeneous base catalyst, biodiesel, transesterification, *Camelina* oil

## Abstract

A novel mesoporous Zn/MgO hexagonal-nano-plate catalyst was synthesized by a simple template-free hydrothermal method and applied in the base-catalyzed transesterification of *Camelina* oil for biodiesel synthesis. The Zn/MgO catalyst calcinated at 873 K exhibited the highest catalytic activity with a yield of 88.7%. This catalytic reaction was performed using 3% *w*/*w* of the catalyst with a methanol-to-oil molar ratio of 24:1 at 393 K in 8 h. The excellent catalytic performance is possibly attributed to its favorable textural features with relatively high surface area (69.1 m^2^ g^−1^) and appropriate size of the mesopores (10.4 nm). In addition, the as-synthesized catalyst demonstrated a greater basic sites density than single mesoporous MgO, which might have been promoted by the addition of Zn, leading to a synergetic interaction that enhanced its catalytic activity. This catalytic system demonstrated high stability for five catalytic runs and catalytic activity with over 84% yield.

## 1. Introduction

Biomass was one of the major sources of energy and fuel supply in the late 1800s; however, lower diesel fuel production costs have retarded its development. The recent concerns about the finite reserves and environmental impact of diesel fuel have driven scientists to explore a cheap and energy-efficient way to produce fuel from biomass, the only natural and renewable carbon resource abundant enough to substitute fossil fuel. Several reports have indicated that the combination of biodiesel with various catalysts improves the combustion behavior of diesel engines and produces great environmental benefits because of the closed carbon cycle and lower exhaust emissions of unburnt hydrocarbons, particulate matter, sulfur oxides, carbon monoxide, etc. [1,2,3,4,5,6]. Moreover, biodiesel possesses similar combustion properties as commercial diesel fuel and thus can be adapted to regular diesel engines without modification of the latter [7,8,9].

Vegetable oils, animal fat, low-valued non-edible oil, and waste lipid can be used as renewable feedstock sources. However, they cannot be used directly as fuel because of their high viscosity and low volatility that cause poor atomization and deposition in the injector in the diesel engine. *Camelina* oil was selected as a potential feedstock for biodiesel synthesis in this study. *Camelina sativa* is an underexploited and low-input crop species of tremendous economic potential. Its seed contains 30 to 40% of oil that is rich in Omega-3 fatty acids [10]. Cold-pressed filtered *Camelina* seed oil produces a maximum power output of 43.35 kW, while that of mineral fuel is only 38.50 kW [11]. It exhibits a positive energy balance, with a net energy ratio of 1.47 in biodiesel production [12]. Moreover, its substantially lower production cost makes it an attractive candidate over other oil crops such as soybean, rapeseed, corn, etc.

Transesterification is a typical organic reaction employed for biodiesel synthesis, in which triglycerides are transformed into mono-alkyl esters through the interchange of the alkoxy moiety with the alcohol molecule in the presence of a catalyst [13]. Alkyl ester products exhibit reduced viscosity and high volatility and can readily be combusted as a fuel. Conventionally, homogeneous acids and bases such as concentrated sulphuric acid (H_2_SO_4_) and sodium hydroxide (NaOH) are employed for biodiesel synthesis. However, feedstock containing high levels of free fatty acids cause saponification and emulsification of biodiesel products.

These problems can be eliminated by switching to the use of heterogeneous catalysts. Calcium oxide (CaO) and magnesium oxide (MgO) were reported in many pioneering works as heterogeneous base catalysts for the transesterification of vegetable oil for biodiesel synthesis [14,15]. However, few studies have been performed because of the relatively low catalytic activity of alkaline earth metal oxides owing to their weak basic strength and high solubility in methanol [16,17]. The modification of MgO chemistry by adding other catalyst species such as metallic ions and oxides to obtain a binary metal oxide system and enhance the catalyst activity and stability has been widely reported [18,19,20]. Extensive studies have revealed that metal-doped MgO demonstrated excellent catalytic activity in various base-catalyzed organic reactions such as propane oxidation [21] and aldol condensation [22]. Metal-doped MgO has also been employed in transesterification for biodiesel synthesis. Dahlquist et al. [23] have successfully synthesized a Li-doped MgO catalyst that gave a high biodiesel yield of 93.9% at 333 K in 2 h. However, significant metal leaching of active species was found, causing catalyst deactivation. Cobalt-doped MgO supplied a high biodiesel yield of 90% in reaction conditions including a methanol-to-oil molar ratio of 9:1, using 5.00 wt.% catalyst at 423 K for 2 h. However, this catalyst demonstrated low catalytic stability, and the biodiesel yield dropped by 50%. It was reported that catalyst deactivation is attributed to catalyst poisoning by organic molecules in the reactant mixture and catalyst leaching of the active species [24]. Solid MgO–ZnO mixed metal oxides were also employed in biodiesel synthesis. It was found that the bi-functional system of Mg and Zn exhibited a synergetic interaction that is effective for enhancing both catalytic activity and stability towards transesterification. Different synthetic methods such as the sol–gel method [25], pulsed-laser deposition [26], and thermal decomposition [27] have been employed for the synthesis of MgO–ZnO mixed metal oxides. However, they usually involve complicated synthetic procedures, comparatively high temperature, and expensive equipment.

In order to extend the lifetime of the MgO catalyst, modification of the chemistry of the MgO catalytic system is needed to decrease its solubility in alcohols and increase its surface basicity. Reinoso and co-workers reported the application of zinc oleate as a catalyst for biodiesel synthesis. A biodiesel yield of 95% was obtained [28]. Our previous studies also reported the development of zinc oxide (ZnO) nano-stars and zinc glycerolates (ZnGly) nano-plates as effective catalysts in simultaneous esterification and transesterification, with excellent biodiesel yield over 95% [29,30]. These results indicate that zinc is a promising active transition metal for catalyzing oil transesterification with different alcohols. Therefore, we considered a bi-functional concept consisting in incorporating Zn^2+^ dopant into the crystal lattice of MgO without changing its original size and morphology. The synergistic effect of a bi-functional system would enhance its catalytic activity and the reusability towards transesterification.

Moreover, solid catalysts encounter mass-transfer limitations to the transesterification system, and their low active sites available for catalytic reaction have limited their application. The development of nano-catalysts with mesoporous structures has improved the situation by increasing the ratio between surface area and volume. Mesoporous structures also increase the bulk particle size, allowing a more effective separation by filtration than nano-sized catalysts.

In the present study, a simple template-free hydrothermal synthesis of a mesoporous Zn/MgO hexagonal nano-plates catalytic system was developed. This catalytic system exhibited a hierarchical combination of interconnected channels, thus minimized the diffusional limitations of the reactants and products. The as-synthesized Zn/MgO catalyst was found highly stable and active towards transesterification with methanol for biodiesel synthesis from *Camelina* oil.

## 2. Materials and Methods

### 2.1. Synthesis of the Catalysts

**Scheme 1:**
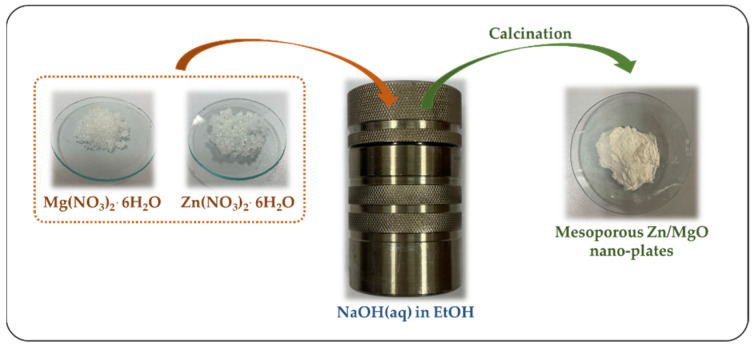
Scheme diagram showing the hydrothermal synthesis of mesoporous Zn/MgO nano-plates catalyst.

Mesoporous Zn/MgO was synthesized through an alkali hydrothermal approach, as shown in Scheme 1. A precursor solution containing Mg(NO_3_)_2_·6H_2_O (513 mg, 2 mmol) and Zn(NO_3_)_2_·6H_2_O (595 mg, 2 mmol) was mixed in an alkaline medium of NaOH(aq) (20 mL, 10 M) and ethanol (20 mL) in a 100 mL Teflon cup inside a stainless steel autoclave. The reaction mixture was heated at 423 K for 24 h under static conditions. The as-synthesized catalyst was filtered out, washed, and then dried in an oven at 383 K for 12 h. The catalyst was then calcinated in the air at designated temperatures of 773 K, 873 K, 973 K, and 1073 K for 3 h. Single mesoporous MgO was also prepared through the same synthetic protocol with the addition of the Mg(NO_3_)_2_·6H_2_O precursor only. No agglomeration was observed during the synthesis of the mesoporous MgO and Zn/MgO catalysts.

### 2.2. Characterization of the Catalysts

The crystal phases of the as-synthesized catalysts were identified by powder X-ray diffraction (XRD) using a Rigaku SmartLab with a CuKα (λ= 1.541862Å) radiation, operated at 45 kV and 200 mA, with a scan rate of 2-theta, ranging from 20° to 80°. The morphology of the as-synthesized catalysts was characterized by a scanning electron microscope (SEM) (Hitachi S4800 FEG SEM system), equipped with energy-dispersive X-ray spectroscopy (EDS) (Horiba EMAX EDS) detectors for elemental analysis, and a transmission electron microscope (TEM) (Philips Tecnai G2 20 S-TWIN TEM system) in conjunction with INCAx-sight EDS detectors for elemental analysis.

Nitrogen adsorption–desorption isotherms of the catalysts were measured on a Micromeritics ASAP 2020 instrument. The catalysts were pre-treated by outgassing under a high vacuum at 350 °C for 6 h before the measurement. The specific surface areas were calculated by the Brunauer–Emmett–Teller (BET) method, while pore size distribution (average pore diameter and pore volume) was calculated according to the Barrett–Joyner–Halenda (BJH) method.

The basic strengths of the samples were determined by the Hammett indicator method. The solid catalyst sample (20 mg) was suspended in a methanol solution of Hammett indicator (1 mL, 0.02 mol/L) and left for 2 h to achieve equilibration. A change from acidic color to basic color was observed if the catalyst possessed higher basic strength than the indicator.

Hammett indicators–benzoic acid titration is a common method for determining the basic strength and the amount of basic sites of a catalyst. Basicity (basic site distribution) of these catalysts was evaluated by the Hammett indicator–benzene carboxylic acid titration method. The catalyst (20 mg) was suspended in methanolic Hammett indicator solution (1 mL, 0.02 mol/L) and left for 2 h. The above mixture was then transferred to a 50 mL Erlenmeyer flask containing methanol (10 mL), followed by titration with benzoic acid in methanol (0.01 M). The end point was noted as the point at which the basic color of the indicator disappeared. Triplicated measurements were done to minimize the end-point color determination error. The basicity is expressed as mmol g^−1^ calculated from the benzoic acid in methanol titrant (0.01 M) needed for the specific amount of catalyst used.

The Hammett indicators employed to study basic strength and basicity were as follows: neutral red (*H*_ = 6.8), bromothymol blue (*H_* = 7.2), phenolphthalein (*H*_ = 9.3), Nile blue (*H*_ = 10.1), Tropaeolin O (*H_* = 11), and 2,4-dinitroaniline (*H_* = 15).

X-ray photoelectron spectroscopy (XPS) studies were also performed using a Kratos Axis Ultra XPS system equipped with monochromatic Al−Kα radiation of 1486.6 eV and with an electron take-off angle of 90°. The pressure of the sample chamber was kept at 10^−8^ Torr during the measurements. The spectrum was recorded in the binding energy (B.E.) range of 0.00 to 1400.00 eV with a step size of 1.00 eV. Energy calibration was performed with the C 1s peak of carbon at 285.0 eV.

### 2.3. Catalytic Reactions and Biodiesel Determination

Catalytic transesterification experiments were carried out in a stirred batch reactor containing the catalyst (66 mg, 3% *w*/*w*, with respect to the weight of oil), oil (2.4 mL), and methanol (2.4 mL, 24:1 MeOH-to-oil molar ratio), and the reaction was heated at the temperature of 393 K for 8 h with a stirring rate of 1000 rpm. After the reaction, the liquid product was isolated from the solid catalyst. The upper layer containing the biodiesel was extracted from glycerol with *n*-hexane. Residue methanol and *n*-hexane were then removed under vacuum. The biodiesel product was re-dissolved in CDCl_3_ for ^1^H NMR analysis with an AV400 Bruker FT-NMR spectrometer. The biodiesel yield was calculated using the signal integral of H of the methoxy group (–OCH_3_) of FAME and that of the α-methylene group (α-CH_2_) of both FAME and oil with the equation below [31].

(1) $$\mathrm{Biodiesel}\;\mathrm{Yield}\;(\%)=\frac{2\;\text{×}\;\mathrm{integral}\;\mathrm{of}\;H\;\mathrm{of}\;{-\mathrm{OCH}}_{3}\;}{3\;\text{×}\;\mathrm{integral}\;\mathrm{of}\;H\;\mathrm{of}\;\alpha -{\mathrm{CH}}_{2}\;}\;\text{×}100\%$$

Reusability studies were carried out with solid catalysts recovered by centrifugation, followed by washing with *n*-hexane and then methanol. The catalyst was dried at 393 K for 5 h and evaluated under the same reaction conditions.

## 3. Results and Discussions

### 3.1. Characterization of the Catalysts

**Figure 1:**
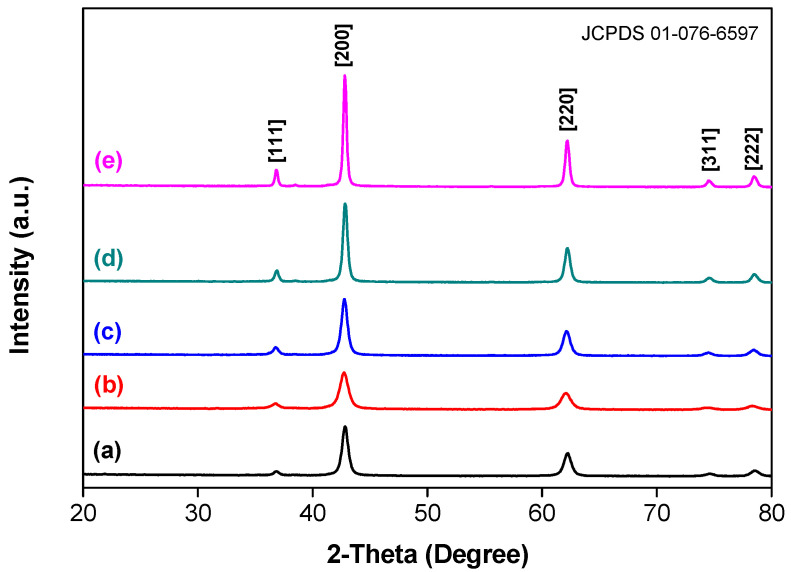
XRD patterns of (**a**) mesoporous MgO calcinated at 873 K and mesoporous Zn/MgO calcinated at (**b**) 773 K, (**c**) 873 K, (**d**) 973 K, and (**e**) 1073 K.

The powder X-ray diffraction (XRD) patterns of mesoporous Zn/MgO calcinated at different temperatures are shown in Figure 1. The XRD patterns of the Zn/MgO catalyst calcinated at 773 K, 873 K, 973 K, and 1073 K showed characteristic diffraction peaks of the single cubic periclase phases (JCPDS 01-076-6597) as the single mesoporous MgO. The absence of characteristic diffraction peaks of ZnO revealed that Zn^2+^ had in art replaced Mg^2+^ in the host lattice, giving a homogeneous solid solution. The close ionic radii of Zn^2+^ (0.74 Å, coordination number CN = 6) and Mg^2+^ (0.72 Å, coordination number CN = 6) favor the easy incorporation of Zn^2+^ into the MgO crystal lattice [32,33,34,35].

**Figure 2:**
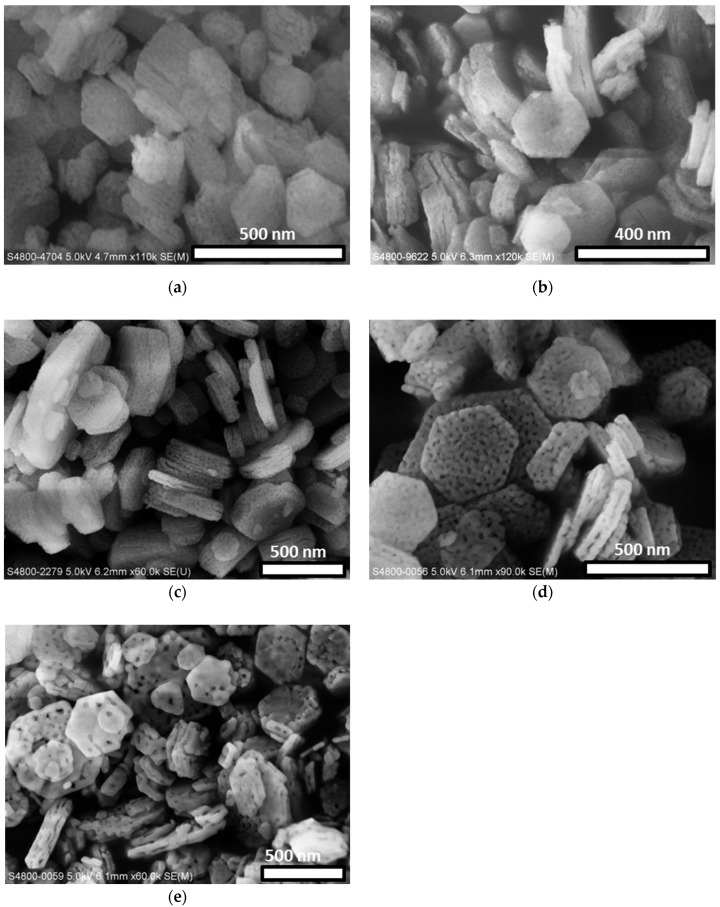
SEM micrographs of mesoporous (**a**) single mesoporous MgO-873 K, (**b**) Zn/MgO-773 K, (**c**) Zn/MgO-873 K, (**d**) Zn/MgO-973 K, and (**e**) Zn/MgO-1073 K.

The surface morphology of mesoporous Zn/MgO calcinated at various temperatures was examined by SEM, and the micrographs are displayed in Figure 2. No agglomeration was observed during the synthesis of the mesoporous MgO and Zn/MgO catalysts. Zn/MgO-773 K (Figure 2b) comprised layers of hexagonal plates with lateral length between 100 to 500 nm and thickness in the range of 50 to 200 nm, with uniform mesopores evenly distributed on the plate surfaces. The formation of such mesoporous Zn/MgO nano-plate is the result of the solvothermal annealing process. The phenomenon was reported for single mesoporous MgO. The hexagonal Mg(OH)_2_ prisms change into 3D wormhole-like mesoporous prisms with the preservation of the hexagonal morphology of Mg(OH)_2_ due to the thermal decomposition process. The solvothermal process leads to the formation of octahedron Mg(OH)_2_, with Mg ions layers separated by two adjacent hydroxyl ions layers. The subsequent calcination process leads to the partial dissociation of the hydrogens bonds associated with the two adjacent hydroxyl ions layers, releasing water molecules and generating the mesopores [36]. The morphology of Zn/MgO was retained when the calcination temperature was raised. However, it could be observed that the pore size increased as the calcination temperature of the samples increased. The pores of Zn/MgO-1073 K (Figure 2e) appeared to be the largest in size when comparing all catalysts, the number of pores was lower, and the pore distribution appeared less orderly on the plate surface. Interestingly, single mesoporous MgO (Figure 2a) also exhibited a similar hexagonal morphology with similar mesoporosity. Therefore, it is likely that Zn ions were incorporated into the MgO crystal lattice without changing the original morphology of MgO.

**Figure 3:**
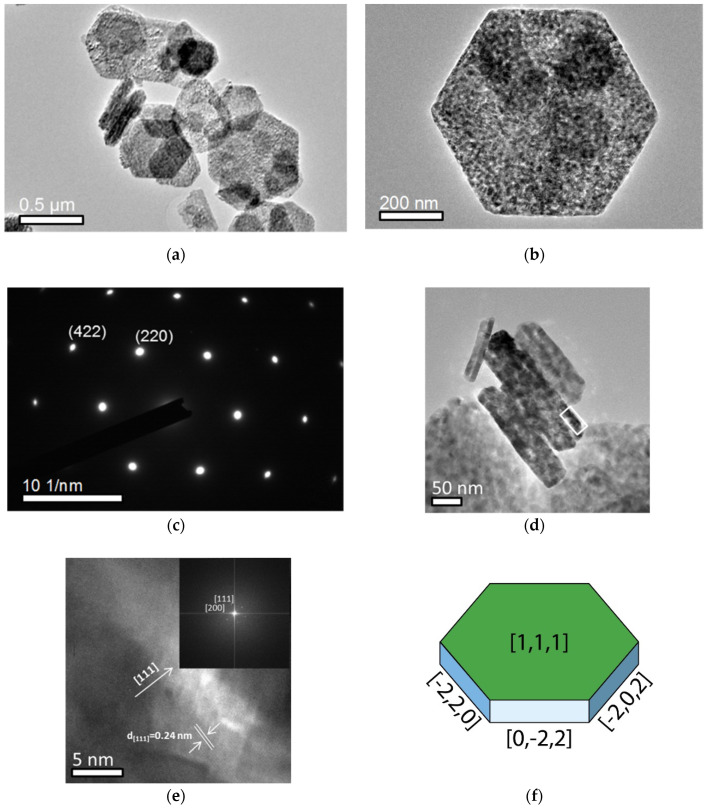
(**a**) Overall TEM micrograph of Zn/MgO-873 K, (**b**) TEM micrograph of the front side of the hexagonal nano-plate and (**c**) corresponding SAED pattern, (**d**) TEM micrograph of the lateral side of the hexagonal nano-plate and (e) corresponding HR-TEM image (insert: FFT image), and (**f**) schematic diagram showing the different facets of the Zn/MgO-873 K nano-plate.

Representative TEM micrographs and the corresponding SAED pattern of Zn/MgO-873 K are displayed in Figure 3. It was observed that the catalyst comprised layers of hexagonal plates with uniform mesopores evenly distributed on the plate surfaces. The SAED pattern (Figure 3c) confirmed the existence of the cubic periclase phase. High-resolution transmission electron microscopy (HR-TEM) and the corresponding fast Fourier transform (FFT) image (Figure 3e) identified the surface termination as exposing polar (111) facets as the main surface on the hexagonal side (Figure 3f), with the generation of low coordination defect sites, which are reported to be highly basic, leading to high catalytic activity [35,37,38].

The average atomic percentage of Zn in Zn/MgO was found to be 7.8% by EDX analysis, which agreed well with the results obtained by inductively coupled plasma mass spectrometry (ICP–MS), with 8.4% of Zn. Furthermore, ICP–MS confirmed the absence of sodium (Na) in the catalysts.

**Figure 4:**
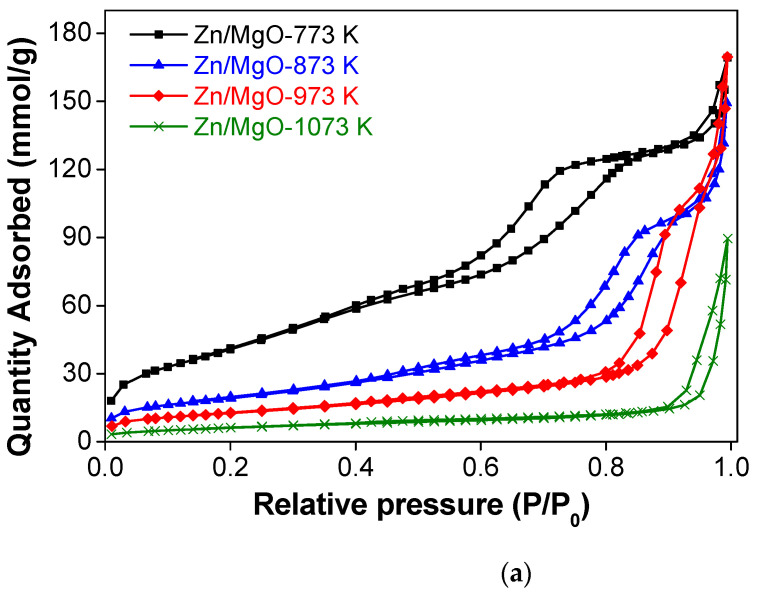

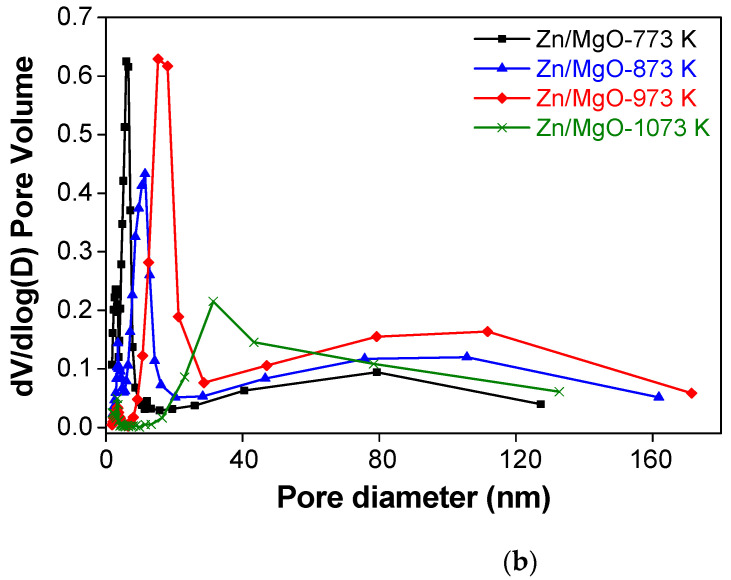
(**a**) N_2_ adsorption–desorption isotherms and (**b**) BJH pore size distribution of mesoporous MgO and Zn/MgO nano-plates calcinated at different temperatures.

The physical properties, such as crystallite size, BET surface area, total pore volume, and pore diameter of all Zn/MgO nano-plates calcinated at different temperatures are summarized in Table 1. The crystallite sizes of Zn/MgO were calculated from the XRD pattern using Scherrer’s equation, and it was found that the crystallite size increased with the increase of the calcination temperature because of the sintering effect [39]. The results were aligned to the results of the BET analysis. The surface areas decreased with the increase in crystallite size. The nitrogen adsorption–desorption isotherms and the pore size distribution of all Zn/MgO catalysts calcinated at a temperature varying from 773 K to 1073 K are displayed in Figure 4a,b respectively. Zn/MgO calcinated at 773 K, 873 K, and 973 K exhibited a type IV isotherm, which is a characteristic of material possessing mesoporosity. Dual hysteresis loops were observed in the samples with an H1 hysteresis loop and an H3 hysteresis loop, which indicated the presence of both mesopores (2 to 50 nm) and macropores (>50 nm) in the samples [40,41]. The hysteresis loop type H1 in the P/P_0_ range between 0.5 and 0.9 indicated the presence of approximately even and uniform mesopores in a reasonably regular array, accompanied by a narrow distribution of pore size. On the other hand, the H3 hysteresis loop in a P/P_0_ range from 0.9 to 1.0 did not clearly show any adsorption isotherm plateau in the P/P_0_ range close to unity, indicating the presence of slit-shaped pores with the Barrett–Joyner–Halenda (BJH) pore size distribution extending to the macropore range. It likely resulted from the aggregation of plate-like particles [40]. Zn/MgO-1073 K showed a type IV isotherm that is a characteristic of material with nonporous or potential macroporous structure and with high energy of adsorption. In addition, Zn/MgO calcinated at 773 K, 873 K, and 973 K possessed a relatively narrow distribution of pore size (Figure 4b), with average pore diameters of 5.4 nm, 10.4 nm, and 18.8 nm, respectively (Table 1). However, the Zn/MgO-1073 K catalyst exhibited a broader pore size distribution with an average pore diameter of 22.7 nm. Interestingly, Zn/MgO-873 K possessed comparable textural properties to those of mesoporous MgO-873 K, as shown in Table 1.

The results of the comparison of basic strength, basic site distribution, and total basicity between all Zn/MgO catalysts and single mesoporous MgO are summarized in Table 2. All Zn/MgO catalysts possessed a basic strength of 9.3 < *H*_ < 10.1 and superior total basicity as compared to single mesoporous MgO. Among all Zn/MgO catalysts, Zn/MgO-873 K revealed the greatest total basicity (1.52 mmol g^−1^), with 0.90 mmol g^−1^, 0.15 mmol g^−1^, and 0.47 mmol g^−1^ basic sites of 6.8 < *H*_, 6.8 < *H*_ < 7.2 and 7.2 < *H*_ < 9.3, respectively. It suggests that at least three types of basic sites exist on the catalyst surface. The remarkably larger number of basic sites in 7.2 < H_ < 9.3 of all Zn/MgO catalysts indicates a higher density of stronger basic sites in comparison with single mesoporous MgO. These findings are well complemented by Olutoye’s studies which have found that the basicity of a catalyst was enhanced by the synergetic interaction between Mg and Zn.

**Figure 5:**
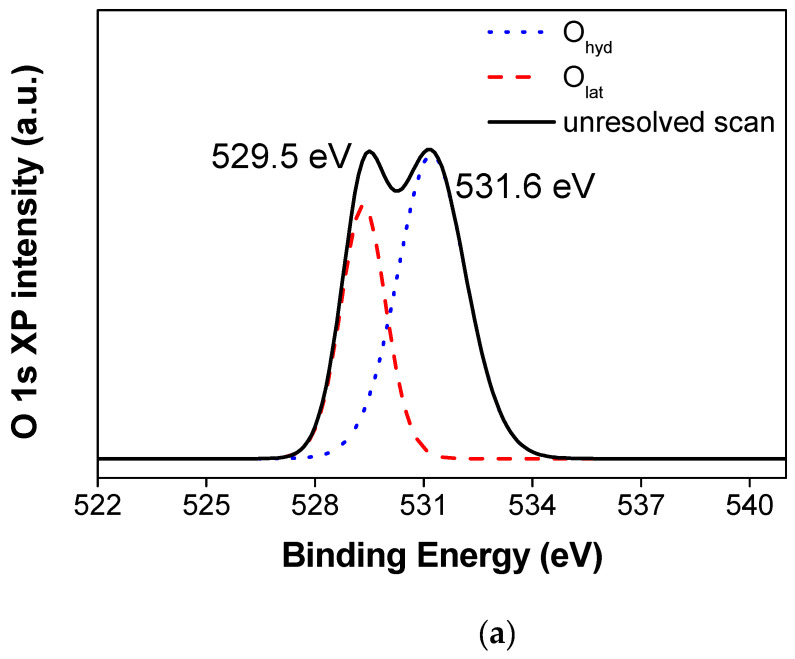

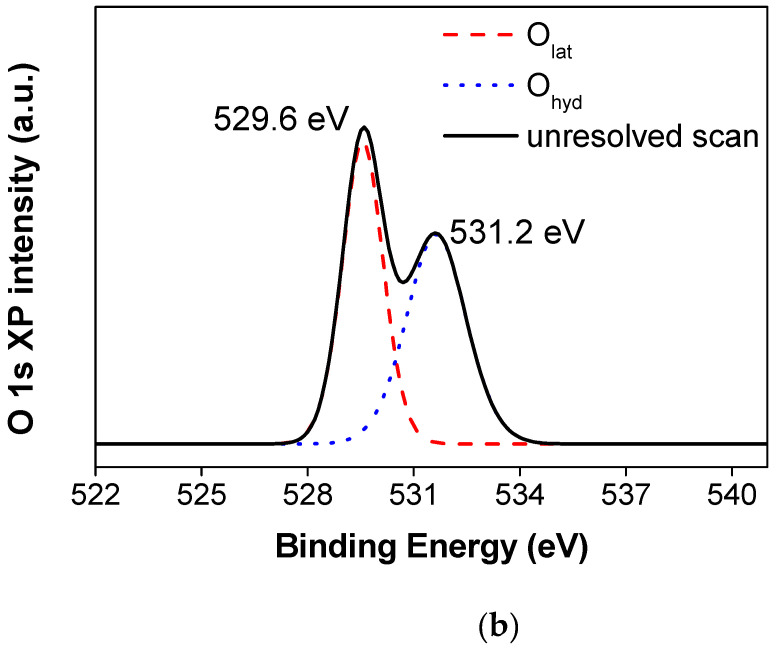
O1s XPS peaks of (**a**) mesoporous MgO and (**b**) Zn/MgO nano-plate catalyst calcinated at 873 K.

The XPS survey data of Mg, Zn, and O elements for Zn/MgO-873 K catalyst compared to those of single MgO and ZnO are depicted in Table 3. The binding energy of 49.3 eV, 1021.1 eV, 529.6 eV, and 531.6 eV corresponds to Mg 2p, Zn2p_3/2_, O_lat_, and O_hyd_, respectively. The slight shift in the Auger parameter of Zn was attributed to the incorporation of Zn^2+^ into the MgO crystal lattice as Zn–O–Mg [42]. The asymmetric O 1s peak of MgO, ZnO, and Zn/MgO was further resolved into the lattice oxygen (O_lat_) and the surface hydroxyl group (O_hyd_) [43]. The peak at lower binding energy was ascribed to lattice oxygen, while the one at higher binding energy was attributed to the hydroxyl group. A lower O 1s binding energy indicates a higher electron pair donation ability and, therefore, a stronger basic strength [44]. Zn/MgO exhibited a slightly higher O_lat_ binding energy than MgO, which might be attributed to the incorporation of Zn^2+^ into the MgO crystal lattice [42]. Lattice oxygen on the catalyst surface serves as a Lewis basic site for transesterification [45]. Figure 5 shows the respective resolved O 1s peaks of mesoporous MgO and Zn/MgO; the surface percentage of lattice oxygen was calculated as summarized in Table 4. It is noted that the Zn/MgO catalyst contained a higher percentage of surface lattice oxygen than MgO. A higher percentage of surface lattice O^2^^−^ indicates a higher number of active Lewis basic sites. The increase in the percentage of surface lattice O^2-^ contrasts with the insignificant increase in the binding energy of O_lat_ (decrease in basic strength). The XPS results aligned well with the data of basicity determination. Therefore, Zn/MgO possesses a comparable basic strength but a substantially higher amount of active surface basic sites than single mesoporous MgO. Based on the results of benzoic titration and XPS analysis, it is interesting to note that there is a direct correlation between the catalytic transesterification activities and the surface O_lat_ concentration of the catalyst.

### 3.2. Catalytic Study

**Figure 6:**
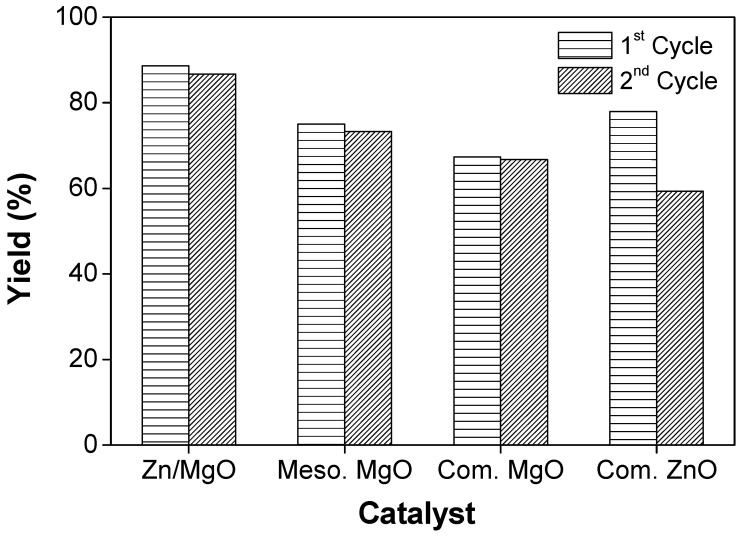
A comparison of catalytic activity and reusability of Zn/MgO with mesoporous MgO, commercial MgO, and ZnO (all calcinated at 873 K). Reaction conditions: catalyst (3% *w*/*w*), MeOH-to-oil molar ratio (24:1), reaction temperature (393 K), and reaction time (8 h).

Catalytic activity and reusability of Zn/MgO nano-plates catalyst were investigated and compared with those of mesoporous MgO, commercial MgO, and ZnO, as depicted in Figure 6. Among all catalysts, Zn/MgO demonstrated the highest catalytic activity and stability, with high biodiesel yields of 88.7% and 86.7% for the first and second run, respectively. Commercial ZnO gave a 78.0% biodiesel yield in the first run, but its catalytic activity decreased drastically to 59.3% in the second reaction cycle. Catalyst deactivation might be due to the leaching of the active species into the reaction medium. Therefore, the high biodiesel yield of ZnO in the first cycle is likely to be due to a homogeneous pathway. Commercial and mesoporous MgO are quite stable in transesterification. Commercial MgO possessed a low BET surface area of 9.0 m^2^ g^−1^, with a relatively low number of active sites available for the catalytic reaction. Mesoporous MgO showed a higher BET surface area (73.1 m^2^ g^−1^) and possesses a mesoporous nature that may favor mass transfer and hence the catalytic transesterification process, leading to a higher biodiesel yield than commercial MgO. Zn/MgO was found to possess a comparable basic strength but a substantially higher number of active surface basic sites than single mesoporous MgO, as indicated by its higher total basicity determined from benzoic titration and an increased percentage of lattice O^2-^, as shown in the XPS analysis. The enhanced catalytic activity of Zn/MgO compared to single mesoporous MgO is attributed to the substitution of Zn^2+^ into the MgO crystal lattice, causing lattice distortion with defects that might occur at corners, edges, and steps, which creates additional sites with ion pairs of low coordination numbers and provides a large active surface for substrates to bind. In addition, threefold-Mg^2+^–threefold-O^2-^ (Mg^2+^_3c_–O^2−^_3c_) was reported to be the most reactive, as it is the most coordinatively unsaturated [38]. In addition, such defects could trap electrons on the surface of crystallites [46]. These might enhance the basicity and activity of the Zn/MgO catalyst. As discussed previously, the surface termination on the lateral side of hexagonal plates of Zn/MgO predominately exposes (111) and (200) facets, with the generation of low coordination defect sites [35,37,38]. The high surface energy polar (111) crystal plane comprises alternating monolayers of cations and anions, which leads to the creation of a strong electrostatic field perpendicular to the polar plane [29] and might enhance the interaction between reactant molecules with the surface. It is concluded that the results demonstrated a high biodiesel yield compared to single mesoporous MgO and commercial MgO and ZnO, proving that the successful incorporation of Zn^2+^ dopant into MgO crystal lattice creates a synergistic effect, attributed to the high catalytic activity of the Zn/MgO catalytic system.

**Figure 7:**
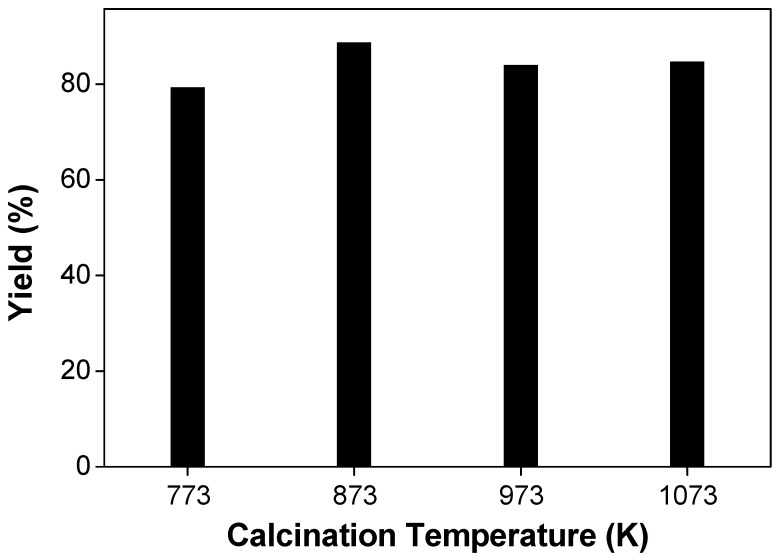
Biodiesel yield of Zn/MgO as a function of the calcination temperature. Reaction conditions: catalyst (3% *w*/*w*), MeOH-to-oil molar ratio (24: 1), reaction temperature (393 K), and reaction time (8 h).

The effect of the calcination temperature on biodiesel conversion by Zn/MgO was studied, and the results are depicted in Figure 7. Zn/MgO-773 K gave the lowest biodiesel yield of 79.3%. Though the catalyst exhibited the largest BET surface area of 151.1 m^2^ g^−1^ as compared to those annealed at higher calcination temperature, its comparatively low average pore diameter (5.4 nm) restricted the accommodation of the bulky triglyceride moiety that possesses a diameter of approximately 5.8 nm [47]. Zn/MgO-873 K possessed a BET surface area with a value smaller than half of Zn/MgO-773 K; however, its larger pore diameter (10.4 nm) favored the accessibility of the triglyceride substrate to the active sites, resulting in an increased biodiesel yield from 79.3% to 88.7%. We observed a slight drop in biodiesel yield with a further increase in the calcination temperature because the decrease in BET surface area counteracted the effect of the increase in pore size. Apart from the textural properties, the surface basicity of the catalysts also significantly contributed to the catalytic activity. All the Zn/MgO catalysts calcinated at various temperatures exhibited an equal surface basic strength of 9.3 < *H_* < 10.1; however, the total basicity was different, with the values of 1.07, 1.52, 1.42, and 1.47 mmol g^−1^ for Zn/MgO calcinated at 773 K, 873 K, 973 K, and 1073 K, respectively. These results align quite well with the trend of the respective catalytic performances in transesterification.

**Figure 8:**
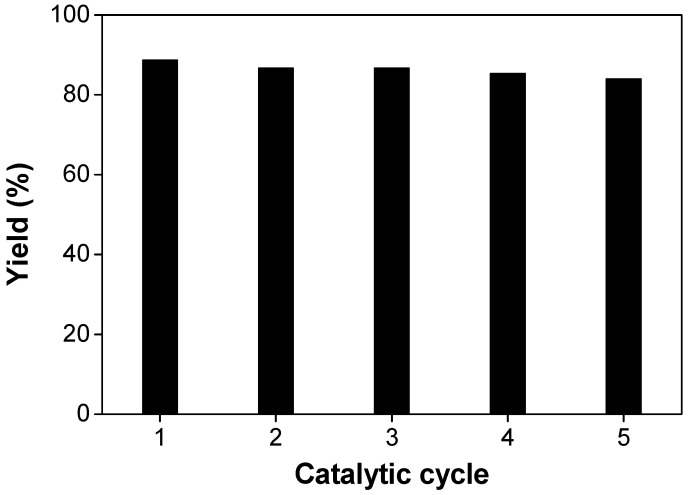
Reusability of Zn/MgO-600 °C. Reaction conditions: catalyst (3% *w*/*w*), MeOH-to-oil molar ratio (24:1), reaction temperature (393 K), and reaction time (8 h).

Catalyst reusability is one of the essential features determining the economic viability for the commercialization of an industrial process. Zn/MgO-873 K, which possessed the highest catalytic activity, was therefore subjected to reusability studies. It was found that the catalytic system had high stability in transesterification, with the biodiesel yield remaining over 84.0% in five catalytic runs, as shown in Figure 8. This high catalytic stability is attributed to the defects induced by the incorporation of Zn^2+^ into the MgO crystal lattice [47]. The reusability of the as-synthesized catalyst makes it advantageous over the homogenous counterpart, as it could lower the overall production cost. To the best of our knowledge, several studies reported the use of MgO–ZnO catalysts for biodiesel synthesis; however, harsh reaction conditions were applied. Olutoye et al. [47] employed Mg_1−x_Zn_1+x_O_2_ in the transesterification of used vegetable cooking oil, with the highest FAME yield of 78% achieved at a high reaction temperature of 461 K. Lee et al. [48] studied the activity of a series of Mg–Zn mixed metal oxides in the transesterification of *Jatropha* oil. The highest biodiesel yield of 83% was achieved for MgO–ZnO at a Mg/Zn atomic ratio of 8 (MZ8), with 3% catalyst dosage and a 24:1 methanol-to-oil molar ratio at 393 K for 3 h. MZ8 exhibited a consistent decrease in activity from 83% to 63% in five consecutive runs of reaction, with an activity retention of 76%. In comparison, our catalysts demonstrated a higher catalytic activity and stability towards transesterification, with the biodiesel yield decreased slightly from 89.7% to 84.0% for Zn/MgO-873 K. An activity retention of 94% was obtained after five successive catalytic runs for Zn/MgO-873 K.

A study of the reaction extent for the as-synthesized Zn/MgO-873 K revealed a slow transesterification with low biodiesel yield at the beginning, which is proposed to be due to the low mass transfer limit within the three phases system. The transesterification proceeded much faster afterwards, reaching a maximum yield at 6 to 8 h. However, the biodiesel yield remained almost the same by extending the reaction time. Due to the excess of methanol used in this transesterification, the overall reaction most likely followed a pseudo-first-order reaction kinetics model, comparable to that of a similar catalytic system we reported [49].

Further development and investigation of this as-synthesized catalytic system during oil transesterification, including massive production of catalyst and large-scale biodiesel synthesis or even pilot-scale continuous flow reactions toward biodiesel synthesis, will be the subject of our future investigations. For large-scale biodiesel production, optimization and kinetic studies are important to analyze the role of each parameter (methanol-to-oil molar ratio, reaction temperature, catalyst loading, and reaction time) and pinpoint the most prominent parameter. This information will help design and adapt our synthesized catalytic system for large-scale biodiesel production. Moreover, the fuel properties of the final biodiesel product produced using the as-synthesized catalytic system in combustion engines are also important to understand whether the biodiesel fuel is associated with a lower emission of toxic gases. These experiments are now ongoing and will be discussed and published in the future.

## 4. Conclusions

A novel mesoporous Zn/MgO hexagonal-nano-plate catalyst was synthesized and demonstrated transesterification of *Camelina* oil, with biodiesel yield of 88.7%. This was achieved at 393 K in 8 h using 3% *w*/*w* of the catalyst with a MeOH-to-oil ratio of 24:1. Its excellent catalytic performance was mainly associated with a relatively high surface area (69.1 m^2^ g^−1^), a pore size of 10.4 nm, and a high total basicity of 1.52 mmol g^−1^, all these values being better than those measured for single MgO. The incorporation of Zn^2+^ into the MgO crystal lattice induced defects that exhibited high catalytic activity and stability for at least five catalytic cycles, with biodiesel yield above 84.0%.

## Figures and Tables

**Table 1:** Physical properties of mesoporous MgO and Zn/MgO nano-plates calcinated at different temperatures.

Catalyst	Crystallite Size ^1^ (nm)	S_BET_ ^2^ (m^2^ g^−1^)	V ^3^ (cm^3^ g^−1^)	D_aver_ ^4^ (nm)
Meso. MgO-873 K	12.6	73.1	0.267	12.0
Zn/MgO-773 K	9.7	151.1	0.269	5.4
Zn/MgO-873 K	12.7	69.1	0.233	10.4
Zn/MgO-973 K	17.8	45.3	0.263	18.8
Zn/MgO-1073 K	24.1	22.6	0.140	22.7

^1^ Calculated from XRD patterns by employing Scherrer’s equation; ^2^ BET surface area; ^3^ Total pore volume; ^4^ Average pore width.

**Table 2:** Surface basicity of mesoporous MgO and Zn/MgO nano-plates catalysts calcinated at different temperatures.

Catalyst	Basic Strength by Hammett Indicator	Basicity by Benzoic Titration (mmol g^−1^)			
		6.8 < *H_*	6.8 < *H_* < 7.2	7.2 < *H_* < 9.3	Total Basicity
MgO-873 K	9.3 < *H_* < 10.1	0.79	0.25	0.01	1.05
Zn/MgO-773 K	9.3 < *H_* < 10.1	0.60	0.15	0.32	1.07
Zn/MgO-873 K	9.3 < *H_* < 10.1	0.90	0.15	0.47	1.52
Zn/MgO-973 K	9.3 < *H_* < 10.1	0.67	0.30	0.45	1.42
Zn/MgO-1073 K	9.3 < *H_* < 10.1	0.59	0.34	0.54	1.47

**Table 3:** Binding energy of Mg^2+^, Zn^2+^, O_lat_, and O_hyd_ of mesoporous MgO, ZnO, and Zn/MgO calcinated at 873 K.

Catalyst	Binding Energy (eV)						
	Mg 2p	Zn 2p_1/2_	Zn 2p_3/2_	Zn LMM	Auger Parameter ^1^	O_lat_	O_hyd_
MgO	49.3	-	-	-	-	529.5	531.2
ZnO	-	1045.2	1022.1	498.9	2009.8	531.0	532.5
Zn/MgO	49.3	1044.2	1021.1	497.7	2010.0	529.6	531.6

^1^ Auger parameter = K.E. (Zn KLMM) + B.E. (Zn 2p_3/2_).

**Table 4:** Surface percentage of O_lat_ of single mesoporous MgO and Zn/MgO calcinated at 873 K.

Catalyst	Surface Percentage of O_lat_ (at.%)
Mesoporous MgO	23.5
Zn/MgO	32.8
